# Supplementary material for: Seminoma and Embryonal Carcinoma Footprints Identified by Analysis of Integrated Genome-Wide Epigenetic and Expression Profiles of Germ Cell Cancer Cell Lines
Source: PLoS One. 2014 Jun 2;9(6):e98330. doi: 10.1371/journal.pone.0098330 (PMC4041891; doi:10.1371/journal.pone.0098330)
Supplement: File S1 — ZIP file containing DMRforPairs output for significant regions. Please start from the html files. (ZIP) [file pone.0098330.s008.zip › figures/21019.pdf]

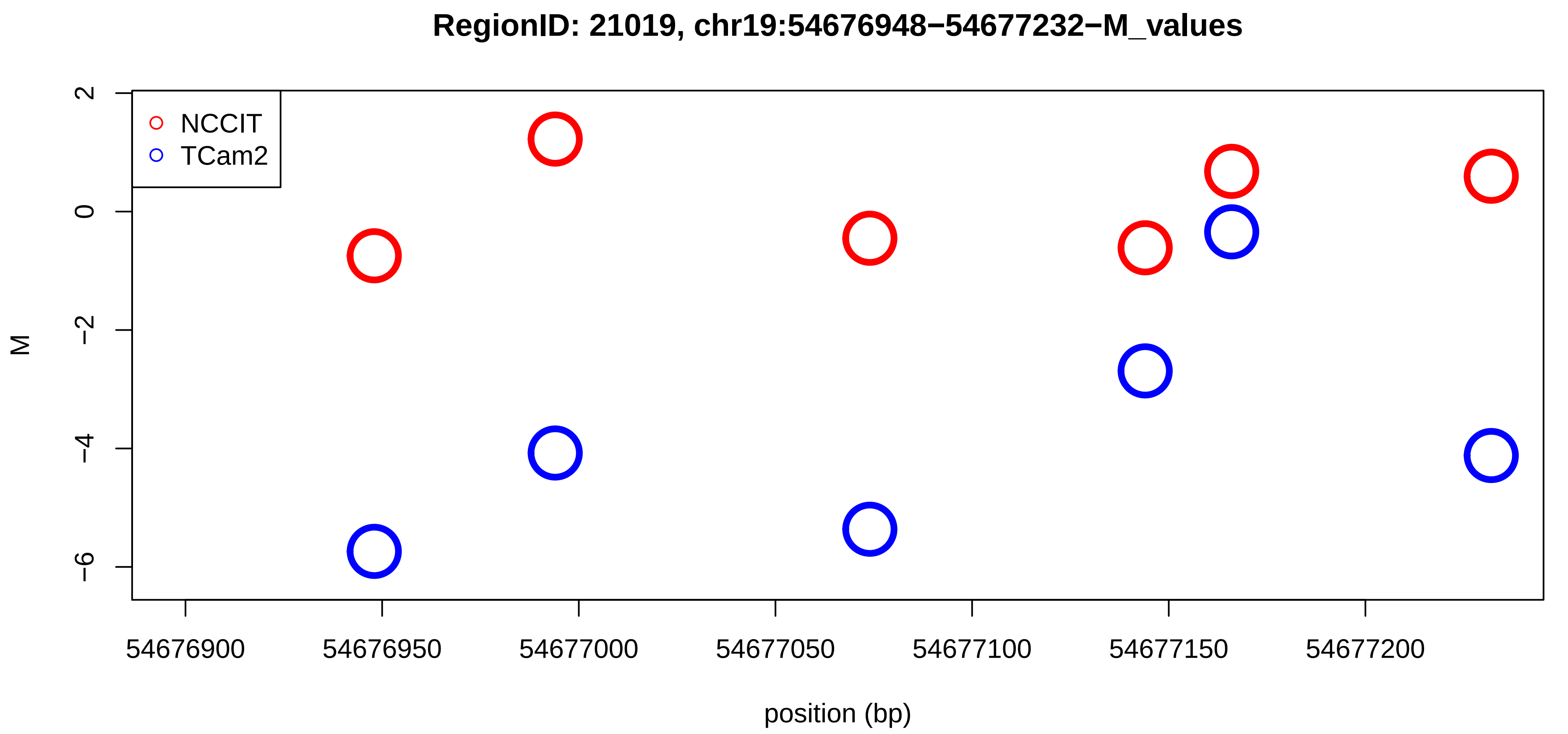
RegionID: 21019, chr19:54676948−54677232−M_values
NCCIT
TCam2
M
2
0
−2
−4
−6
54676900
54676950
54677000
54677050
54677100
54677150
54677200
position (bp)

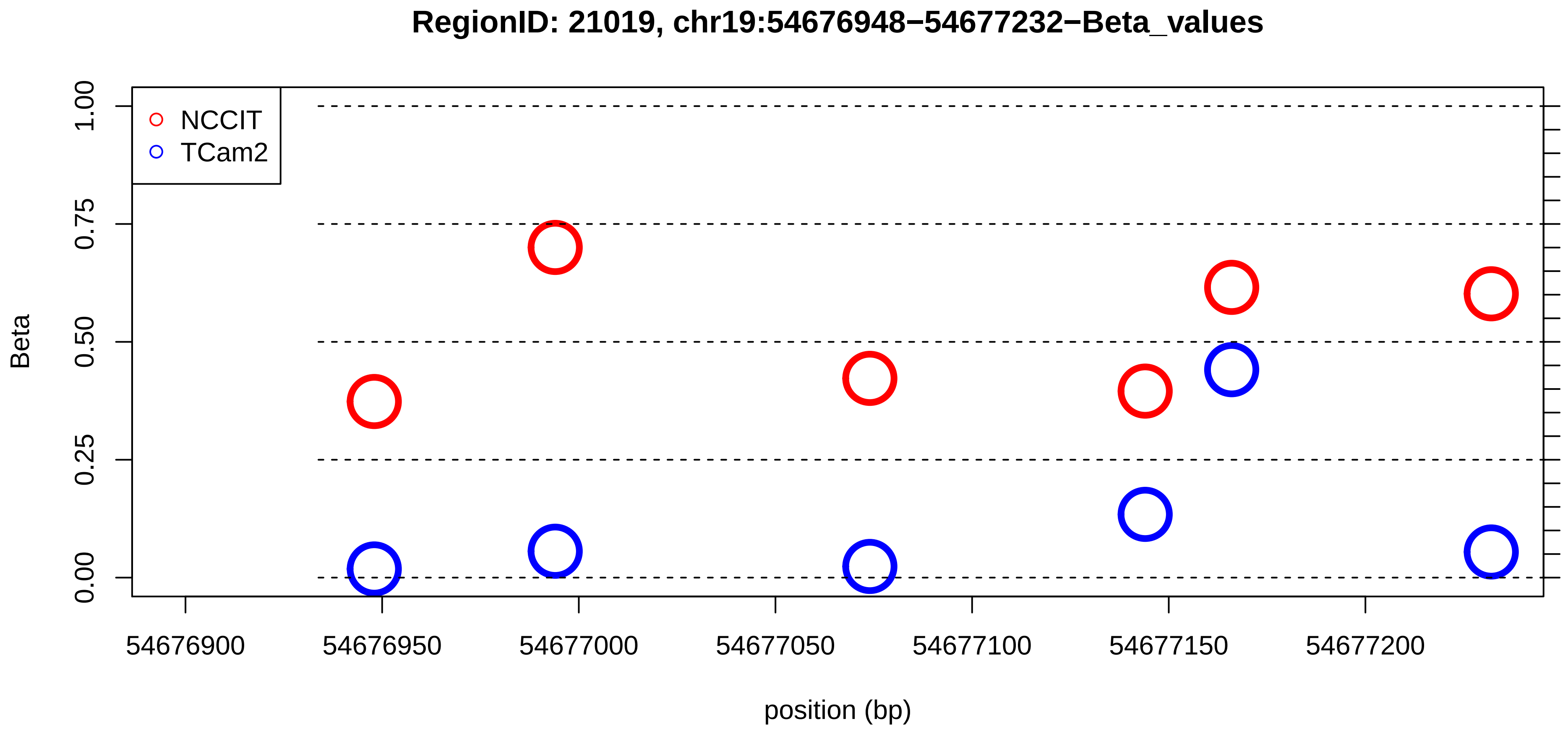
RegionID: 21019, chr19:54676948−54677232−Beta_values
NCCIT
TCam2
Beta
1.00
0.75
0.50
0.25
0.00
54676900
54676950
54677000
54677050
54677100
54677150
54677200
position (bp)
